# A Unified Deep Learning Framework for Instance Segmentation Across Diverse Cytological Stains

**DOI:** 10.1111/cyt.70087

**Published:** 2026-05-11

**Authors:** Luís Otávio Santos, Rodrigo de Paula E. Silva Ribeiro, Bibiana Quatrin Tiellet, Laura Maria Effting Silva, Fabiana Botelho de Miranda Onofre, Alexandre Sherlley Casimiro Onofre, Aldo von Wangenheim

**Affiliations:** ^1^ Department of Computer Sciences Federal University of Santa Catarina Florianopolis Brazil; ^2^ Department of Clinical Analysis Federal University of Santa Catarina Florianopolis Brazil; ^3^ National Institute for Digital Convergence (INCoD) Federal University of Santa Catarina Florianopolis Brazil

**Keywords:** computer‐assisted, cytopathology, deep learning, image processing, instance segmentation, whole slide imaging

## Abstract

**Objective:**

Assess whether a single instance‐segmentation model can operate robustly across multiple cytological stains, avoiding stain‐specific pipelines without sacrificing accuracy.

**Methods:**

We consolidated three expert‐annotated datasets (Papanicolaou, Feulgen, AgNOR), standardised their formats and trained Mask R‐CNN, Mask2Former, YOLO11n and YOLO12n under two regimes: per‐stain and unified multi‐stain. We reported AP_50_ and AP_75_ on validation/test to decouple detection tolerance from boundary precision, using identical training and evaluation protocols across models.

**Results:**

Feulgen specialist models yielded the highest scores in our benchmark, with YOLO11n reaching AP_75_ 0.566 and AP_50_ 0.643. Mask2Former led in geometric precision (AP_75_) in complex scenarios (AgNOR and Papanicolaou), achieving the best performance on the combined dataset. Crucially, unified training matched or exceeded stain‐specific models, maintaining detection (AP_50_) while improving boundary definition (AP_75_).

**Conclusions:**

A single multi‐stain model is feasible for cytology instance segmentation, preserving detection and improving boundary accuracy in diverse stains. Transformer‐based Mask2Former showed the best AP_75_ on the combined dataset, supporting a unified multi‐stain, scalable path to AI‐assisted cytopathology and practical screening workflows.

**Inside This Month's Cytopathology:**

Can a single computer vision model handle the diversity of cytological stains found in clinical routine? This study validates a unified deep learning approach that effectively segments cells across Papanicolaou, Feulgen and AgNOR protocols. Results suggest that laboratories can deploy simplified, stain‐invariant screening tools without compromising diagnostic precision, overcoming a major barrier in digital pathology interoperability.

## Graphical abstract

Transformer‐based unified cytology segmentation across Papanicolaou, Feulgen and AgNOR achieves stain‐invariant performance. Mask2Former maximises boundary precision (AP75) on the combined dataset, enabling one model to replace multiple stain‐specific deployments without accuracy loss while simplifying clinical integration.

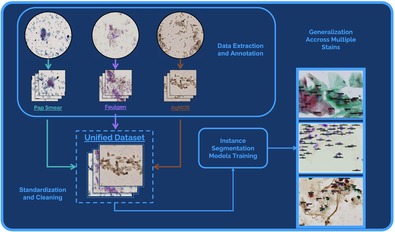

## 1 Introduction

Cytopathology is essential for screening neoplastic and infectious diseases, particularly in the cervix and oral cavity [1, 2]. However, routine assessment still relies on expert visual interpretation, which is time‐consuming and requires experienced pathologists [3]. Observer variability and fatigue further affect consistency, especially when subtle morphological features challenge interpretation [4, 5]. Automated high‐throughput approaches with standardised precision offer a promising alternative to enhance diagnostic accuracy and efficiency [3].

Instance segmentation has emerged as a key application of computer vision in biomedical image analysis, benefiting from recent breakthroughs in model architecture and learning strategies. Architectures such as Mask2Former [6], Mask R‐CNN [7] and modern YOLO variants [8, 9] have demonstrated robust performance in delineating complex structures.

Recent studies have started to explore unified or stain‐generalizable models trained on multi‐stain datasets as viable alternatives to multiple stain‐specific models [10, 11]. Approaches utilising meta‐learning and domain‐aware learning have demonstrated that extracting stain‐invariant representations allows for robust generalisation across different histological protocols with minimal fine‐tuning [10, 11, 12, 13].

While commercial whole slide imaging ecosystems have successfully integrated automated screening tools, these solutions often utilise proprietary formats that challenge interoperability and are optimised for a narrow range of routine stains, which creates a barrier for implementing adaptable AI tools in heterogeneous workflows [14, 15].

## 2 Objectives

Current stain‐specific pipelines fragment training data and complicate deployment. We investigate whether unified multi‐stain training delivers competitive segmentation under realistic screening constraints across diverse protocols (Papanicolaou, AgNOR and Feulgen).

### 2.1 Research Question

Can a single instance‐segmentation model be trained to perform robustly across multiple cytological staining protocols?

### 2.2 Research Objectives

1. Quantify the effect of unified multi‐stain training versus per‐stain training across diverse architectures.
2. Assess boundary quality and detection robustness under heterogeneous chromatic distributions typical of cytology.
3. Analyse accuracy‐latency trade‐offs relevant to practical screening and triage.

## 3 Contributions

To answer the research question and align with the objectives, we present a practical multi‐stain study and toolkit for cytology instance segmentation. We treat cross‐stain variability as a training signal rather than a nuisance factor, as it will be described in the following sections.

- *Unified multi‐stain segmentation*: We demonstrate that a single instance segmentation model can be effectively trained to handle cytological images stained with diverse protocols (Papanicolaou, Feulgen, AgNOR), overcoming the traditional need for stain‐specific models.
- *Comprehensive benchmarking*: We provide a systematic comparison of state‐of‐the‐art convolutional and transformer‐based segmentation architectures (Mask R‐CNN, Mask2Former, YOLO11n, YOLO12n) on both individual and combined multi‐stain cytopathology datasets.
- *Generalisation and scalability*: Unified training maintains or improves segmentation accuracy across stains, suggesting a scalable approach for robust AI‐assisted cytopathology in heterogeneous clinical environments.
- *Open multi‐stain resource*: We consolidate and publicly document three annotated cytology datasets with standardised protocols, supporting future research on generalizable medical image segmentation.

## 4 Materials and Methods

To evaluate model generalisation across cytological protocols, we consolidated three expert‐annotated datasets: Papanicolaou (OCPap), AgNOR (CCAgT) and Feulgen. Together, these unified resources encompass over 20,000 image patches from oral and cervical anatomical sites.

### 4.1 Datasets

The data used in this study were collected from subjects at the Hospital Universitário Professor Polydoro Ernani de São Thiago (HU/UFSC). Papanicolaou and AgNOR slides were digitised using an Axio Scan.Z1 slide scanner (Zeiss) equipped with a Hitachi HV‐F202SCL camera, generating Whole Slide Images (WSIs) with a spatial resolution of 0.111 μm/pixel. Feulgen slides were captured using a Motic BA410 microscope coupled with a Motic Pro 285A camera.

**FIGURE 1:**
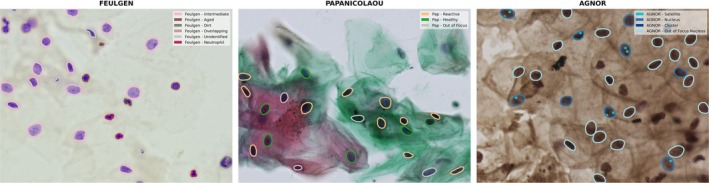
Samples from each dataset (Feulgen, Papanicolaou and AgNOR).

Papanicolaou (OCPap) [16] comprises 9797 image patches (1200 × 1600) from oral cytology. Annotations cover seven classes, focusing on nuclear distinctions between healthy, reactive and abnormal epithelial cells. AgNOR (CCAgT) [17] consists of 9339 patches (1600 × 1200) from cervical cytology. This dataset targets nucleolar organiser regions (NORs), with seven classes differentiating nuclei, clusters and satellites. Feulgen contains 1955 patches (1200 × 1600) from cervical samples stained for DNA quantification. Eight classes were annotated, capturing variations in chromatin density and cell ageing. The biological characteristics of the annotated classes for each stain are detailed in Table 1. To illustrate the morphological diversity and annotation standards described above, Figure 1 presents representative patches from the Feulgen, Papanicolaou and AgNOR datasets, displaying the ground truth masks for the varying nuclear and cellular structures.

**TABLE 1:** Description of annotated classes across Papanicolaou, AgNOR and Feulgen datasets.

Stain	Class	Description
Papanicolaou	Healthy	Nucleus from healthy epithelial cell.
	Out of focus	Nucleus that appears blurred due to imaging artefact.
	Blood cells	Nucleus from blood cell, often considered contaminants.
	Abnormal	Nucleus from an epithelial cell exhibiting irregular morphology.
	Reactive cell	Nucleus from a cell undergoing inflammatory or reparative processes.
	Dividing	Nucleus undergoing mitosis, characterised by condensed chromatin.
	Not defined	Nucleus or structure that cannot be clearly classified.
AgNOR	Nucleus	A cellular nucleus, appearing as a rounded region in brown shades.
	Cluster	Atypical NORs, non‐rounded structures (kidney‐shaped or aggregated dots) or large black dots always located within a nucleus.
	Satellite	Typical NORs, small rounded black dots always located inside a nucleus.
	Out of focus	Nucleus that appears blurred due to focus issues, making NOR identification impossible.
	Overlapped	Nuclei where it is not possible to determine which nucleus the NOR belongs to.
	Non‐viable	Object visually resembling a nucleus in colour, texture or shape, but considered an artefact to be ignored in WSI analysis.
	Leukocyte	Nuclei from white blood cells (leukocytes).
Feulgen	Altered	Nuclei exhibiting atypical morphological features or chromatin patterns, which may indicate dysplasia or malignant transformation.
	Intermediate	Nuclei from intermediate squamous epithelial cells, representing a normal cytological class with moderate nuclear size and chromatin density.
	Aged	Nuclei from superficial epithelial cells that have undergone ageing or keratinisation, often showing pyknotic or condensed chromatin.
	Dirt	Small particles or debris mistakenly detected as nuclei, including dust, staining artefacts or extraneous material.
	Overlapping	Regions where two or more nuclei overlap, making clear segmentation or classification challenging.
	Unidentified	Nuclei or structures that could not be reliably classified into any of the defined categories, either due to image quality or ambiguous morphology.
	Neutrophil	Nuclei from neutrophil granulocytes (a type of white blood cell), typically multilobulated and distinct from epithelial nuclei.
	Spot	Small, darkly stained nuclear fragments or spots, often corresponding to apoptotic bodies, micronuclei or artefacts.

#### 4.1.1 Dataset Curation and Expert Validation

We curated high‐fidelity masks through a rigorous pipeline involving a multidisciplinary team of senior cytopathologists. To ensure consistency across protocols, we employed a standardised workflow using Labelme [18] and Labelbox [19]. The encompassing four critical phases are:

- *ROI selection*: Pathologists screened WSIs to select regions of interest (ROIs) containing clinically significant cellular representations;
- *Extraction*: Selected ROIs were cropped at full resolution to preserve morphological details;
- *Annotation*: Specialists delineated nuclear and subnuclear structure using polygon tools;
- *Review*: All annotations underwent a review process; ambiguities and non‐validated annotations were discarded.

This labour‐intensive process ensured a high‐quality baseline. Average annotation time was approximately 10 min per patch. The development of the Papanicolaou and AgNOR datasets each spanned 14 months. The Feulgen dataset followed the same protocol but was completed in 8 months due to limited availability. Our annotation team comprised PhD candidates, professors and an undergraduate student undergoing specialised training. To ensure quality and consistency, images underwent a secondary review by our experienced team members.

#### 4.1.2 Combined Dataset

Prior to model training, we implemented a thorough data curation pipeline. Images without valid annotations (i.e., empty segmentation arrays) were removed and floating‐point annotation values were rounded down to the nearest integer. Additionally, misclassified annotations were manually corrected with specialist's support. Finally, the cleaned datasets were merged and converted into both COCO and YOLO formats. We named this set as the Multi‐Cyto [20] dataset. Table 2 shows the distribution of all classes over the training, validation and test sets. Total number of images is 17,422 and total number of annotations is 119,874. The total number of samples in training, validation and testing are 12,502 (71.76%), 2448 (14.05%) and 2472 (14.19%).

**TABLE 2:** Dataset distribution of annotations across staining protocols, classes and dataset splits.

Stain	Category name	Training set		Validation set		Test set	
		Annotations (*n*)	Annotations (%)	Annotations (n)	Annotations (%)	Annotations (n)	Annotations (%)
AgNOR	Nucleus	9799	11.3	2142	13.1	2072	12.6
	Cluster	17,832	20.5	4014	24.5	3864	23.5
	Satellite	4638	5.3	884	5.4	956	5.8
	Out of focus	6520	7.5	1423	8.7	1327	8.1
	Overlapped	978	1.1	194	1.2	201	1.2
	Non‐viable nucleus	1018	1.2	207	1.3	187	1.1
	Leukocyte nucleus	3455	4.0	724	4.4	755	4.6
Feulgen	Altered	2178	2.5	218	1.3	218	1.3
	Intermediate	5210	6.0	639	3.9	615	3.7
	Aged	2254	2.6	276	1.7	265	1.6
	Dirt	430	0.5	66	0.4	67	0.4
	Overlapping	1620	1.9	169	1.0	193	1.2
	Unidentified	4953	5.7	628	3.8	606	3.7
	Neutrophil	3866	4.4	450	2.7	405	2.5
	Spot	1078	1.2	128	0.8	128	0.8
Pap smear	Healthy	1349	1.5	232	1.4	249	1.5
	Out of focus	8180	9.4	1688	10.3	1853	11.3
	Blood	2969	3.4	641	3.9	674	4.1
	Abnormal	8375	9.6	1543	9.4	1693	10.3
	Dividing	44	0.1	4	0.0	5	0.0
	Not defined	63	0.1	9	0.1	9	0.1
	Reactive	245	0.3	85	0.5	114	0.7
All	Total	87,054	72.6	16,364	13.7	16,456	13.7

Our experiment was then conducted using 28 annotated WSIs, comprising 8 stained with Papanicolaou, 5 with Feulgen and 15 with AgNOR. To prevent data leakage, we avoid assigning patches from the same patient to the same split.

### 4.2 Models

To assess segmentation performance across different deep learning‐based segmentation paradigms, we selected four instance segmentation architectures: Mask2Former [6], Mask R‐CNN [7], YOLO11n [8] and YOLO12n [9].

- *Mask2Former*: A single‐stage transformer‐based architecture that employs masked attention to capture global context. It excels at modelling complex boundaries and semantic relationships in crowded scenes, such as densely packed cells.
- *Mask R‐CNN*: A two‐stage CNN‐based architecture. It first generates region proposals using a region proposal network (RPN) before predicting precise pixel‐level masks, effectively handling overlapping objects and defining boundaries.
- *YOLO11n*: A lightweight, single‐stage architecture designed for high throughput. It predicts probabilities and segmentation simultaneously in a single forward pass, prioritising speed and low latency for real‐time scenarios.
- *YOLO12n*: An evolution of the single‐stage paradigm integrating attention modules into the YOLO framework. It enhances feature extraction within a compact structure, maintaining focus on efficient inference.

### 4.3 Experimental Design and Training Details

**FIGURE 2:**
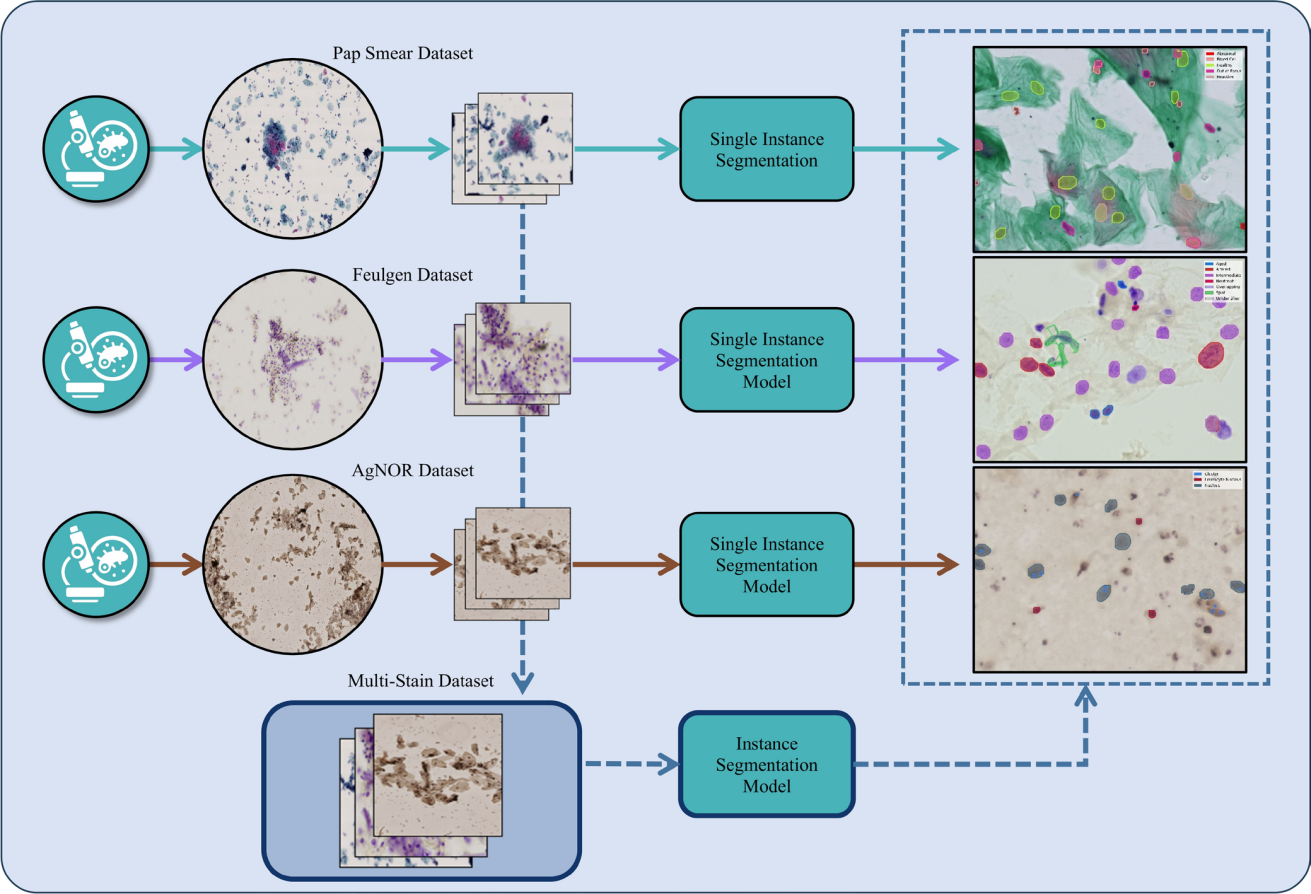
Workflow comparing single‐stain models with a unified multi‐stain model across Papanicolaou, Feulgen and AgNOR.

The overall workflow to evaluate cross‐stain generalisation comprised two distinct phases, as illustrated in Figure 2. Initially, each model was trained and validated independently on three individual staining datasets. Subsequently, these datasets were merged to form a unified Multi‐Cyto dataset to examine whether a single model could generalise effectively across diverse staining types.

This strategy enables a comprehensive assessment of each architecture's robustness and capacity to generalise across varying cytological staining conditions.

We applied the OneCycle [21] learning rate policy across all models to optimise convergence. Mask R‐CNN and Mask2Former (Detectron2) were trained for 100 epochs with an initial learning rate of 0.0001. Configurations are provided in Table 3. All experiments were performed on a NVIDIA DGX H100 server [22].

**TABLE 3:** Training configuration for each model.

Model	Batch	Architecture	Backbone	Optimizer	Pre‐trained
MaskRCNN	30	CNN	ResNet50	SGD	ImageNet + COCO
Mask2Former	20	Transformer	Swin‐B	AdamW	COCO
YOLO11	160	Hybrid	YOLO11‐n	AdamW	COCO
YOLO12	120	Hybrid	YOLO12‐n	AdamW	None

Our work proposes a unified analysis pipeline that integrates multiple staining protocols, aiming to assess whether a single model can generalise effectively across heterogeneous staining types while preserving segmentation accuracy.

## 5 Results

Performance was evaluated using AP_50_ (detection) and AP_75_ (boundary precision) across single and combined multi‐stain datasets. Table 4 presents the validation and test results for all configurations. Feulgen samples yielded the highest scores in this benchmark, led by YOLO11n. AgNOR and Papanicolaou stains presented greater complexity due to dense artefacts. Mask2Former stood out for its geometric precision, achieving AP_75_ in AgNOR (0.367), Papanicolaou (0.327) and notably in combined Multi‐Cyto dataset (0.436). Crucially, the results demonstrate that unified training on the combined dataset consistently matched or exceeded the performance of the stain‐specific specialists at AP_50_ and produced gains at AP_75_ when compared with stain specialists' models.

**TABLE 4:** Instance segmentation performance (AP50 and AP75) comparison between specialist and unified models.

Model	Metric	Pap smear		Feulgen		AgNOR		Combined	
		AP75	AP50	AP75	AP50	AP75	AP50	AP75	AP50
Mask R‐CNN	Validation	0.302	0.325	0.481	0.580	0.313	0.331	0.372	0.430
	Test	0.302	0.323	0.527	0.625	0.308	0.333	0.381	0.433
Mask2Former	Validation	0.331	0.354	0.430	0.550	0.365	0.455	0.431	0.507
	Test	0.327	0.349	0.446	0.554	0.367	0.461	0.436	0.512
YOLO11n‐seg	Validation	0.278	0.332	0.544	0.635	0.275	0.389	0.406	0.492
	Test	0.268	0.318	0.566	0.643	0.268	0.378	0.409	0.500
YOLO12n‐seg	Validation	0.239	0.293	0.263	0.409	0.183	0.269	0.302	0.381
	Test	0.237	0.292	0.298	0.437	0.179	0.268	0.299	0.387

### 5.1 Computational Efficiency

To evaluate operational feasibility in high‐throughput scenarios, such as patient screening or WSI analysis, we conducted inference efficiency test analysis (Table 5). For this experiment, we performed inference on the testing set of each model to each dataset, including the combined. Mask2Former, for instance, proved to be less suitable for real‐time applications due to its lower throughput (15.0 FPS). In contrast, YOLO11n demonstrated the highest throughput (110 FPS) and lowest latency among the tested models.

**TABLE 5:** Computational efficiency analysis comparing inference throughput (FPS), latency and VRAM usage.

Model	Unified			AgNOR			Feulgen			Papanicolaou		
	FPS	Latency	VRAM	FPS	Latency	VRAM	FPS	Latency	VRAM	FPS	Latency	VRAM
Mask2Former	15.0	66.76 ± 5.49	5234	15.1	66.42 ± 6.33	5230	15.0	66.66 ± 6.47	5230	15.3	65.49 ± 5.36	5230
Mask R‐CNN	37.5	26.68 ± 6.11	2847	37.0	27.06 ± 11.96	2797	37.2	26.85 ± 6.26	2823	36.6	27.34 ± 10.31	2848
YOLO12n‐seg	100.0	10.00 ± 6.84	188	101.1	9.89 ± 5.03	153	93.0	10.75 ± 1.98	169	94.6	10.57 ± 10.01	121
YOLO11n‐seg	110.8	9.03 ± 8.61	343	125.6	7.96 ± 5.49	153	110.9	9.01 ± 12.08	225	110.7	9.03 ± 12.00	177

### 5.2 Qualitative Assessment

**FIGURE 3:**
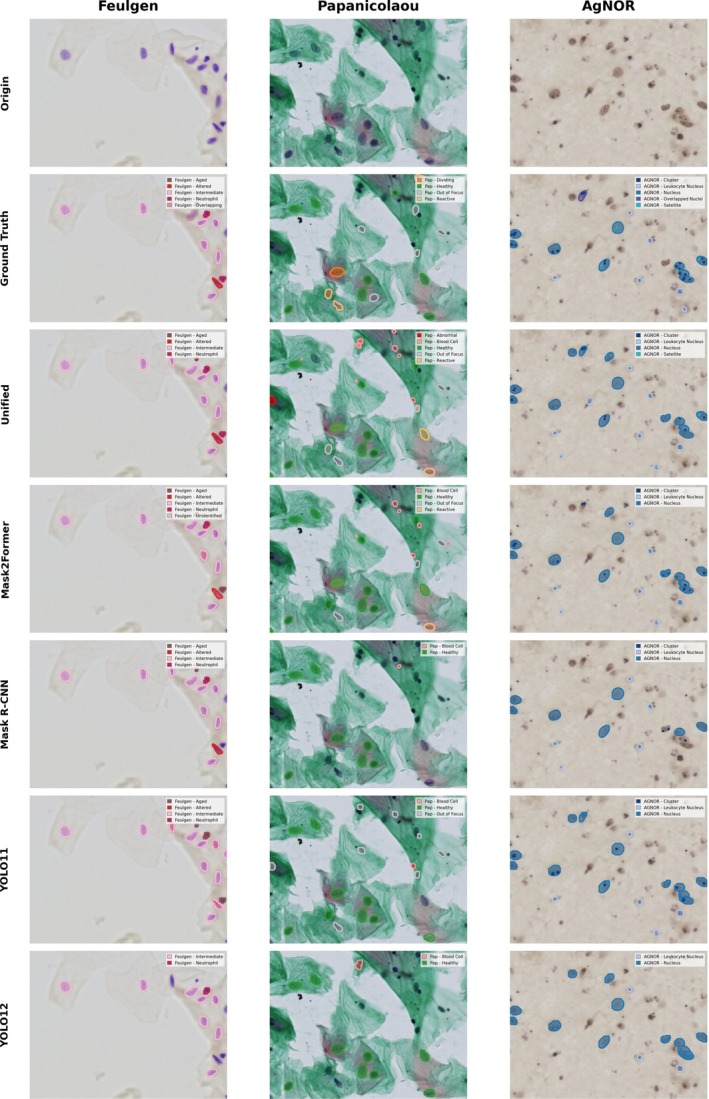
Qualitative comparison of instance segmentation performance across Feulgen, AgNOR and Papanicolaou stains.

As illustrated in Figure 3, visual inspection confirmed that the unified approach produced segmentation quality comparable to specialist models, accurately resolving small clusters and overlapping artefacts. The Unified row (Mask2Former) stood out for identifying small NORs in AgNOR samples, whereas Mask R‐CNN and YOLO12 occasionally failed or merged adjacent signals.

## 6 Discussion

Our results demonstrate that unified multi‐stain trained on the Multi‐Cyto dataset can match or surpass stain‐specific baselines. It particularly improves boundary precision (AP_75_) while preserving detection sensitivity (AP50). These findings confirm the feasibility of a stain‐invariant approach, effectively streamlining digital pathology workflows.

The selected models represent distinct computational paradigms. YOLO11n offered the best speed‐accuracy trade‐off, while YOLO12n underperformed, likely reflecting a need for extensive pre‐training [8]. Mask2Former consistently delivered the highest geometric precision (AP_75_), leveraging global attention mechanisms to model complex boundaries, despite being the slowest when compared to the other models.

### 6.1 Stain Diversity as Regularisation

We selected a representative triad spanning distinct biochemical targets and chromatic protocols to reflect real‐world heterogeneity. Although standard cytopathology diagnostics typically include Papanicolaou, haematoxylin and eosin (H&E) and Romanowsky‐type stains [23, 24], variability in staining protocols remains a major barrier for AI automation [14]. Our selection serves as a rigorous domain generalisation stress test. Papanicolaou [25] provides routine morphological contrast, Feulgen [26] offers stoichiometric DNA quantification and AgNOR [27, 28] introduces challenging granular patterns. Training across these targets, we forced the models to learn structural and spatial features rather than stain‐specific colour patterns, suggesting that cross‐stain variability acts as an implicit regulariser [11].

### 6.2 Relevance to Molecular Cytology

The inclusion of AgNOR addresses a specific computational challenge, as the dense patterns of AgNORs morphologically resemble signals seen in in situ hybridisation (ISH) assays, such as fluorescence (FISH) and chromogenic (CISH), which are increasingly applied to cytological specimens for biomarker testing [29, 30]. From a computer vision perspective, successfully segmenting AgNOR clusters establishes a proof‐of‐concept for algorithms aimed at quantifying molecular signals in digital cytopathology. Therefore, our unified approach demonstrates a robust foundation capable of extending not only routine stains like H&E but also complex molecular quantification tasks [30].

### 6.3 Digital Workflow and Operational Integration

The practical deployment of AI in cytopathology still faces challenges related to interoperability within established digital pathology ecosystems. Many platforms utilise proprietary formats (e.g., .czi, .svs, isyntax) which can create silos, complicating the seamless integration of third‐party or custom AI algorithms [3, 15]. These interoperability constraints underscore the need for vendor‐agnostic and open‐source systems [14], which are designed to enable not only WSI visualisation but also AI development and integration across vendor formats.

## 7 Limitations

While these findings provide valuable insights, some limitations should be considered:

- *Class imbalance*: Uneven distribution of classes within the datasets may lead to biassed model performance, particularly, for under‐represented categories. Collecting more annotations from new slides and employing techniques such as cost‐sensitive learning could help mitigate these effects;
- *Dataset expansion and diversity*: Current dataset's limited scope may restrict the model's ability to generalise across varied staining protocols and clinical settings. Future research should aim to expand the dataset by incorporating additional samples from multiple institutions and a broader range of histopathology staining techniques.

## 8 Conclusions

In this study, we demonstrate that a single instance segmentation model can effectively generalise across multiple cytological protocols. We trained on Papanicolaou, Feulgen and AgNOR datasets; the unified approach matches the segmentation performance of stain‐specific models while simplifying deployment in heterogeneous clinical environments.

Our benchmarking reveals that transformer‐based architectures, particularly Mask2Former, consistently deliver superior boundary precision (AP_75_), critical for cytopathological quantitative analysis.

Key insights from our work include:

- Unified models reduce implementation complexity while maintaining performance across stains (Mask2Former AP_75_: 0.367 AgNOR, 0.446 Feulgen, 0.327 Papanicolaou).
- Transformer architectures outperform CNN and hybrid models in geometric accuracy, with Mask2Former achieving the highest AP_75_ 0.436 on combined multi‐stain data.
- Multi‐stain training improves generalisation without compromising stain‐specific performance.

## Author Contributions

Conceptualisation and Programming: Luís Otávio Santos, Rodrigo de Paula E. Silva Ribeiro and Aldo von Wangenheim. Methodology: Luís Otávio Santos and Rodrigo de Paula E. Silva Ribeiro. Validation: Laura Maria Effting Silva, Fabiana Botelho de Miranda Onofre, Alexandre Sherlley Casimiro Onofre and Bibiana Quatrin Tiellet. Data curation: Luís Otávio Santos, Rodrigo de Paula E. Silva Ribeiro, Laura Maria Effting Silva, Fabiana Botelho de Miranda Onofre, Alexandre Sherlley Casimiro Onofre and Bibiana Quatrin Tiellet. Writing – review and editing: All authors. Supervision: Aldo von Wangenheim.

## Funding

This work was financed in part by Coordenação de Aperfeiçoamento de Pessoal de Nível Superior—Brasil (CAPES)—Finance Code 001.

## Ethics Statement

All datasets used in this study were collected in accordance with the guidelines of Research Ethics Committee at the Federal University of Santa Catarina (CEPSH/UFSC). Papanicolaou dataset was approved under protocol number 23193719.5.0000.0121. Both AgNOR and Feulgen datasets were approved under protocol number 57423616.3.0000.0121. In all cases, participants (or their legal guardians) provided written informed patient consent and all procedures were conducted in accordance with the Declaration of Helsinki and relevant local regulations.

## Conflicts of Interest

The authors declare no conflicts of interest.

## Data Availability

The Multi‐Cyto dataset used in this study is openly available at Mendeley Data: https://data.mendeley.com/datasets/b8bygdxr7r/1. Source code to reproduce all experiments, figures and metrics is available at: https://github.com/LuisOtavioSantos/MultCyto‐UFSC. Additional materials (model weights, configs and scripts) are included in the repository.

## References

[1] E. T. H. Fontham , A. M. D. Wolf , T. R. Church , et al., “Cervical Cancer Screening for Individuals at Average Risk: 2020 Guideline Update From the American Cancer Society,” CA: A Cancer Journal for Clinicians 70, no. 5 (2020): 321–346, 10.3322/caac.21628. 32729638

[2] A. H. Alsarraf , O. Kujan , and C. S. Farah , “The Utility of Oral Brush Cytology in the Early Detection of Oral Cancer and Oral Potentially Malignant Disorders: A Systematic Review,” Journal of Oral Pathology & Medicine 47, no. 2 (2018): 104–116, 10.1111/jop.12660. 29130527

[3] M. A. VandeHaar , H. Al‐Asi , F. Doganay , et al., “Challenges and Opportunities in Cytopathology Artificial Intelligence,” Bioengineering 12, no. 2 (2025): 176, 10.3390/bioengineering12020176. 40001695 PMC11851434

[4] I. Kholová , G. Negri , M. Nasioutziki , et al., “Inter‐ and Intraobserver Agreement in Whole‐Slide Digital ThinPrep Samples of Low‐Grade Squamous Lesions of the Cervix Uteri With Known High‐Risk HPV Status: A Multicentric International Study,” Cancer Cytopathology 130, no. 12 (2022): 939–948. 35833701 10.1002/cncy.22624 PMC10084192

[5] R. Maung , “Pathologists' Workload and Patient Safety,” Diagnostic Histopathology 22, no. 8 (2016): 283–287, 10.1016/j.mpdhp.2016.07.004.

[6] B. Cheng , I. Misra , A. G. Schwing , A. Kirillov , and R. Girdhar , “Masked‐Attention Mask Transformer for Universal Image Segmentation,” in 2022 IEEE/CVF Conference on Computer Vision and Pattern Recognition (CVPR), ed. K. Dana , G. Hua , S. Roth , et al. (IEEE Computer Society, 2022), 1280–1289, 10.1109/CVPR52688.2022.00135.

[7] L. Rettenberger , F. R. Münke , R. Bruch , and M. Reischl , “Mask R‐CNN Outperforms U‐Net in Instance Segmentation for Overlapping Cells,” Current Directions in Biomedical Engineering 9, no. 1 (2023): 335–338, 10.1515/cdbme-2023-1084.

[8] R. Khanam and M. Hussain , “YOLOv11: An Overview of the Key Architectural Enhancements,” preprint, arxiv, 2024, https://arxiv.org/abs/2410.17725.

[9] A. MAR and M. Hussain , “YOLOv12: A Breakdown of the Key Architectural Features,” preprint, arxiv, 2025, https://arxiv.org/abs/2502.14740.

[10] R. D. Bülow , J. Kers , and P. Boor , “Multistain Segmentation of Renal Histology: First Steps Toward Artificial Intelligence–Augmented Digital Nephropathology,” Kidney International 99, no. 1 (2021): 17–19, 10.1016/j.kint.2020.08.025. 33390226

[11] A. Konwer and P. Prasanna , “MetaStain: Stain‐Generalizable Meta‐Learning for Cell Segmentation and Classification With Limited Exemplars,” in Medical Image Computing and Computer Assisted Intervention–MICCAI 2024: 27th International Conference, Marrakesh, Morocco, October 6–10, 2024, Proceedings, Part IV. Springer‐Verlag, 2024:307–317, 10.1007/978-3-031-72083-3_29.

[12] O. Pina and V. Vilaplana , “Unsupervised Domain Adaptation for Multi‐Stain Cell Detection in Breast Cancer With Transformers,” in 2024 IEEE/CVF Conference on Computer Vision and Pattern Recognition Workshops (CVPRW), ed. A. Dantcheva , A. Owens , A. Shrivastava , et al. (Institute of Electrical and Electronics Engineers, 2024), 5066–5074, 10.1109/CVPRW63382.2024.00513.

[13] K. Xie , R. Guo , C. Cong , M. Pagnucco , and Y. Song , “Domain Generalised Cell Nuclei Segmentation in Histopathology Images Using Domain‐Aware Curriculum Learning and Colour‐Perceived Meta Learning,” in Frontiers in Artificial Intelligence and Applications, ed. U. Endriss , F. S. Melo , K. Bach , et al. (IOS Press, 2024), 10.3233/FAIA240508.

[14] C. Jansen , B. Lindequist , K. Strohmenger , et al., “The Vendor‐Agnostic EMPAIA Platform for Integrating AI Applications Into Digital Pathology Infrastructures,” Future Generation Computer Systems 140 (2023): 209–224, 10.1016/j.future.2022.10.025.

[15] M. D. Herrmann , D. A. Clunie , A. Fedorov , et al., “Implementing the DICOM Standard for Digital Pathology,” Journal of Pathology Informatics 9 (2018): 37, 10.4103/jpi.jpi_42_18. 30533276 PMC6236926

[16] A. V. Matias , UFSC OCPap: Papanicolaou Stained Oral Cytology Dataset (v4) (Mendeley Data, 2022), 10.17632/DR7YDY9XBK.2.

[17] J. G. A. Amorim , CCAgT: Images of Cervical Cells With AgNOR Stain Technique (Mendeley Data, 2022), 10.17632/WG4BPM33HJ.2.

[18] K. Wada , “labelme: Image Polygonal Annotation With Python,” 2016, https://github.com/wkentaro/labelme.

[19] Labelbox , The Data Factory for AI Teams (Labelbox, 2025), https://labelbox.com.

[20] L. O. Santos , R. Ribeiro , B. Q. Tiellet , et al., UFSC Multi‐Cyto: Multi‐Stain Cytopathology Image Dataset, vol. 1 (Mendeley Data, 2025), 10.17632/b8bygdxr7r.1.

[21] L. N. Smith and N. Topin , “Super‐Convergence: Very Fast Training of Neural Networks Using Large Learning Rates,” in Artificial Intelligence and Machine Learning for Multi‐Domain Operations Applications, ed. T. Pham . vol. 11006 (SPIE, 2019), 369–386, 10.1117/12.2520589.

[22] NVIDIA Corporation , NVIDIA DGX H100/H200 System User Guide (NVIDIA Corporation, 2026).

[23] E. Jörundsson , J. H. Lumsden , and R. M. Jacobs , “Rapid Staining Techniques in Cytopathology: A Review and Comparison of Modified Protocols for Hematoxylin and Eosin, Papanicolaou and Romanowsky Stains,” Veterinary Clinical Pathology 28, no. 3 (1999): 100–108, 10.1111/j.1939-165x.1999.tb01057.x. 12075519

[24] F. M. A. Elmahdi , T. I. Alrfaie , E. M. Alsubaihi , et al., “Comparative Study of Conventional Staining Techniques in Cytology,” International Journal of Science and Research (IJSR) 11, no. 5 (2022): 876–879, 10.21275/SR22514114506.

[25] M. Kamal , “Pap Smear Collection and Preparation: Key Points,” CytoJournal 19 (2022): 24. 35510105 10.25259/CMAS_03_05_2021 PMC9063692

[26] M. Biggiogera , M. Cavallo , and C. Casali , “A Brief History of the Feulgen Reaction,” Histochemistry and Cell Biology 162, no. 1 (2024): 3–12. 38609528 10.1007/s00418-024-02279-9 PMC11227455

[27] L. E. Lindner , “Improvements in the Silver‐Staining Technique for Nucleolar Organizer Regions (AgNOR),” Journal of Histochemistry and Cytochemistry 41, no. 3 (1993): 439–445, 10.1177/41.3.8429207. 8429207

[28] D. Trerè , “AgNOR Staining and Quantification,” Micron 31, no. 2 (2000): 127–131, 10.1016/S0968-4328(99)00069-4. 10588058

[29] S. Savic and L. Bubendorf , “Common Fluorescence In Situ Hybridization Applications in Cytology,” Archives of Pathology & Laboratory Medicine 140, no. 12 (2016): 1323–1330, 10.5858/arpa.2016-0202-RA. 27479335

[30] Z. U. Rehman , M. F. Ahmad Fauzi , W. S. H. M. Wan Ahmad , et al., “Review of In Situ Hybridization (ISH) Stain Images Using Computational Techniques,” Diagnostics 14, no. 18 (2024): 2089, 10.3390/diagnostics14182089. 39335767 PMC11430898

